# Age-Related Phenotypes Linked to Aberrant Expression and Localization of a Telomeric Protein

**DOI:** 10.1093/geroni/igab046.2498

**Published:** 2021-12-17

**Authors:** Amanda Stock, Kun Wang, Chengyu Liu, Ross McDevitt, Chongkui Sun, Yi Gong, Yie Liu

**Affiliations:** 1 National Institute on Aging, Baltimore, Maryland, United States; 2 National Heart, Lung, and Blood Institute, Bethesda, Maryland, United States

## Abstract

Telomere attrition is associated with telomere biology disorders and age-related diseases. In telomere biology disorders, telomere uncapping induces a DNA damage response that evokes cell death or senescence. However, a causal mechanism for telomere attrition in age-related diseases remains elusive. Telomere capping and integrity are maintained by shelterin, a six-protein complex. Rap1 is the only shelterin member that is not required for telomere capping and is expressed at non-telomeric genomic and cytosolic regions. The objective of this study was to determine aberrant phenotypes attributed to non-telomeric Rap1. To test this, we generated a Rap1 mutant knock-in (KI) mouse model using CRISPR/Cas9 editing, in which Rap1 at telomeres is prevented, leaving only non-telomeric Rap1. Cell fractionation/western blotting of primary fibroblasts from Rap1 KI mice demonstrated decreased Rap1 expression and Rap1 re-localization off telomeres, with an altered cellular distribution. This same difference in Rap1 is also observed in human cells with telomere erosion, indicating that aberrant Rap1 in our model may recapitulate Rap1 in aging and human telomere biology disorders. Compared to wild-type control mice, Rap1 KI mice exhibited increased body weight, altered cytokine levels, behavioral deficits, and decreased lifespan. In conclusion, our results reveal a novel mechanism by which telomere shortening may contribute to age-related pathologies by disrupting Rap1 expression and cell localization.

